# Light-In-Flight Imaging by a Silicon Image Sensor: Toward the Theoretical Highest Frame Rate

**DOI:** 10.3390/s19102247

**Published:** 2019-05-15

**Authors:** Takeharu Goji Etoh, Tomoo Okinaka, Yasuhide Takano, Kohsei Takehara, Hitoshi Nakano, Kazuhiro Shimonomura, Taeko Ando, Nguyen Ngo, Yoshinari Kamakura, Vu Truong Son Dao, Anh Quang Nguyen, Edoardo Charbon, Chao Zhang, Piet De Moor, Paul Goetschalckx, Luc Haspeslagh

**Affiliations:** 1 School of Science and Engineering, Kindai University, 3-4-1 Kowakae, Higahsi-Osaka, Osaka 577-8502, Japan; tokinaka@civileng.kindai.ac.jp (T.O.); takano@civileng.kindai.ac.jp (Y.T.); takehara@civileng.kindai.ac.jp (K.T.); hitoshi@ele.kindai.ac.jp (H.N.); 2 School of Science and Engineering, Ritsumeikan University, 1-1-1 Noji-Higashi, Kusatsu, Shiga 525-8577, Japan; skazu@fc.ritsumei.ac.jp (K.S.); tando@fc.ritsumei.ac.jp (T.A.); ngohoainguyen@outlook.com (N.N.); 3 School of Engineering, Osaka University, 1-1 Yamada-oka, Suita, Osaka 565-0871, Japan; kamakura@si.eei.eng.osaka-u.ac.jp; 4 Department of Industrial and Systems Engineering, International University, Vietnam National University HCMC, Linh Trung Ward, Thu Duc District, Ho Chi Minh City 700000, Vietnam; dvtruongson@gmail.com; 5 School of Electronics and Telecommunications, Hanoi University of Science and technology, 1 Dai Co Viet, Bach Khoa, Hai Ba Trung, Hanoi 100803, Vietnam; quang.nguyenanh@hust.edu.vn; 6 Advanced Quantum Architecture Laboratory, EPFL, Rue de la Maladiere 71b, CH-2002 Neuchatel 2, Switzerland; e.charbon@tudelft.nl; 7 Faculty of Engineering, Mathematics and Computer Science, Delft University of Technology, Mekelweg 4, Delft, 2628 CD, The Netherlands; c.zhang-10@tudelft.nl; 8 IMEC, Kapeldreef 75, 3001 Heverlee, Belgium; Piet.DeMoor@imec.be (P.D.M.); Paul.Goetschalckx@imec.be (P.G.); Luc.Haspeslagh@imec.be (L.H.)

**Keywords:** light-in-flight, theoretical temporal resolution limit, ultra-high-speed image sensor

## Abstract

Light in flight was captured by a single shot of a newly developed backside-illuminated multi-collection-gate image sensor at a frame interval of 10 ns without high-speed gating devices such as a streak camera or post data processes. This paper reports the achievement and further evolution of the image sensor toward the theoretical temporal resolution limit of 11.1 ps derived by the authors. The theoretical analysis revealed the conditions to minimize the temporal resolution. Simulations show that the image sensor designed following the specified conditions and fabricated by existing technology will achieve a frame interval of 50 ps. The sensor, 200 times faster than our latest sensor will innovate advanced analytical apparatuses using time-of-flight or lifetime measurements, such as imaging TOF-MS, FLIM, pulse neutron tomography, PET, LIDAR, and more, beyond these known applications.

## 1. Introduction

Since the Abramson’s holographic “Light-in-Flight imaging” in 1978 [1], various technologies have been created to address this attractive topic. Kubota et al. expanded the holographic technology and showed many impressive images of propagating light, such as light progressing in a zigzag manner in a glass plate by total reflection at the upper and the lower glass surfaces [2], light refracted by a triangular prism [3], and even a flying Chinese letter meaning “light” in a three-dimensional space [4]. Gao et al., introduced the paradigm of compressed sensing into a streak camera to catch flying light in 2D [5]. The image of propagating light is spatially encoded with a random, binary pattern by a digital mirror device (DMD) followed by being captured by a streak camera with a widely-opened entrance port. Solving the inverse problem of the above image formation process produces a sequence of flying light images. Liang et al. further improved the technology [6]. Gariepy et al. used a SPAD array of 32 × 32 pixels. Each SPAD pixel can detect a single photon. However, the SPAD array works only under scarcity of light, and the single image of the SPAD looks like polka dots. They repetitively captured a short-pulse laser beam. A post data process is applied to the captured images to reproduce a light-in-flight image [7]. Velten et al. showed propagating light around objects, using a streak camera that can capture one line of the video at a time with picosecond time resolution. The streak camera was synchronized with a pulsed light source scanning the scene over multiple pulses to capture a set of videos of different scan lines of the scene. Then, the scan-line images were stitched to obtain one high-quality video image [8]. 

The backside-illuminated multi-collection-gate image sensor (BSI MCG image sensor) was proposed for ultra-high-speed imaging by silicon image sensors [9,10,11,12]. The test sensor was fabricated by 130-nm CCD-in-CMOS technology developed by imec. The frame interval is 10 ns with the frame count of 10 frames and the pixel count of about 300 kpixels [13]. The fill factor is 100%. High-resolution images of flying light are captured by a single shot with a silicon image sensor for the first time.

When the spatial resolution of optical devices had no longer improved, Rayleigh presented an expression of the spatial resolution limit [14]. With respect to temporal resolution, the authors theoretically derived an approximate expression of the temporal resolution limit of photoelectron conversion layers [15]. For silicon image sensors receiving incident light of 550 nm, the limit is 11.1 ps. The accuracy of the approximate expression had been confirmed by Monte Carlo simulations [15]. In this paper, the strict expression of the temporal resolution is presented and numerically calculated. The results almost perfectly agree with our approximate expression.

The theoretical analysis may appear irrelevant to practical high-speed imaging technology, since the record frame interval of our new image sensor, 10 ns, is still 1000 times longer than the theoretical temporal resolution limit of 11.1 ps. However, theoretical analyses not only provide deep insights into the underlying physics, but also offer valuable tips in developing practical products. The detailed analysis on the temporal resolution revealed the conditions to minimize the resolution [15,16]. By taking these conditions into account, the minimum temporal resolution might be improved up to 50 ps with currently available technologies at the cost of the fill factor [13]. It took 27 years to increase the frame rate from 4500 fps of historic KODAK EKTAPRO HS4540 developed by Etoh in 1991 [17] to 100 Mfps (equivalent to 10 ns). The frame rate has increased, 22,222 times. We have only 200 times (10 ns/50 ps) more to achieve.

Successful light-in-flight imaging will highlight the significance of the theoretical analysis and accelerate development of such ultra-high-speed image sensors. Image sensors operating at 100 ps or less will promote significant innovations in various advanced scientific apparatuses based on lifetime and time-of-flight measurements, such as imaging TOF MS, pulse neutron tomography, LIDAR, and FLIM.

This paper shows (1) a single-shot imaging of flying light by a silicon image sensor with the BSI MCG structure, (2) very high accuracy of the approximate expression of the theoretical highest frame rate derived by the authors in comparison with numerical calculation results of the strict formulation, and (3) further modification of the structure toward the theoretical highest frame rate, keeping the 100% fill factor, by introducing a convex silicon pyramid array. 

## 2. Light-in-Flight Captured by a Single Shot with a Silicon Image Sensor

### 2.1. BSI MCG Image Sensor 

**Figure 1:**
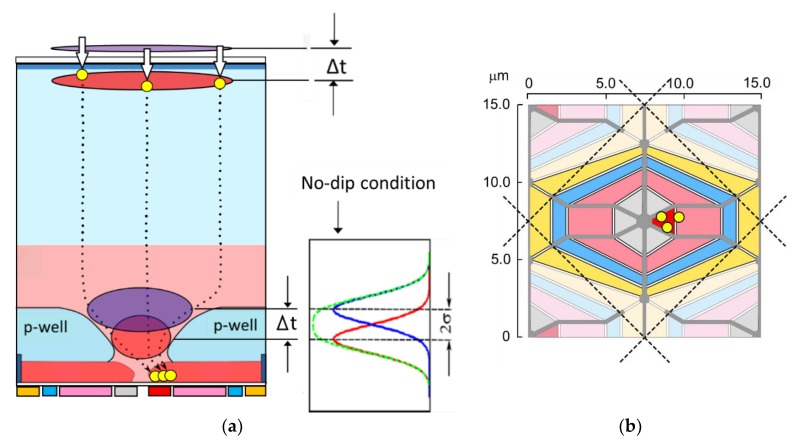
One pixel of a backside-illuminated multi-collection-gate (BSI MCG) image sensor (**a**) A cross section (a pair of instantaneous illuminations with a time difference Δt generate two electron groups. If Δt > 2σ, a dip appears at the center of the superposed distributions of arrival times of the two electron groups, where σ is the standard deviation of the arrival time of one electron group. The non-dip condition is employed to define the temporal resolution limit.) (**b**) Structural pixel area (shown with the configuration of the colored electrodes) and an optical pixel area (surrounded by the dashed lines). Electrodes; grey, collection gates; red, collecting gate (one of the collection gates with a high voltage VH); pink, storage gates; blue, barrier gates; yellow, transfer gates for readout. Optical pixel area: along the dashed lines, the p-well is deepest and the concentration is highest. The p-well creates a linear built-in potential toward the center of each optical pixel. Therefore, electrons generated in the area are guided to the center, and captured by the collecting gate.

Figure 1 shows one pixel of a BSI MCG image sensor which was used in the light-in-flight imaging [12,13]: (a) a cross-section, and (b) an electrode layout on the front side. A p-well with a center hole is created to separate potentials on the upper signal generation layer and the lower diffusion layer for the circuits. The p-well is deepest along the dashed lines in Figure 1b. Therefore, electrons generated by light incident to the area surrounded with the dashed lines are collected to the center hole of the p-well, which defines the optical one pixel area. In Figure 1a, the definition of the temporal resolution limit, Δt = 2σ, is also depicted, where σ is the standard deviation of the arrival time. The definition is used in the theoretical analysis later. 

The structure of the sensor is characterized as follows:

(1) A backside-illuminated image sensor with the fill factor of 100%;
(2) The BSI structure with a p-well prevents electrons generated in the upper layer above the p-well from migrating to the circuits in the lower layer on the front side [9,10];
(3) The built-in linear potential over the p-well toward the center hole is created, which minimizes the travel time of electrons to the center [12,13,18,19];
(4) The silicon layer is 28 μm thick, which absorbs more than 99.9% of incident light with the wavelength less than 650 nm, preventing the remaining light after the absorption from directly intruding into the circuits on the front side and generating false signal electrons there.

Table 1 shows the specification of the sensor. The sensor achieved a temporal resolution of 10 ns. The detailed explanation of the sensor is described in [13].

Image sensors of the sub-nanosecond time resolution for the one-dimensional space have been fabricated [20]. The presented camera with the developed image senor is one of the fastest 2D multi-framing cameras.

### 2.2. Experimental Setup

**Figure 2:**
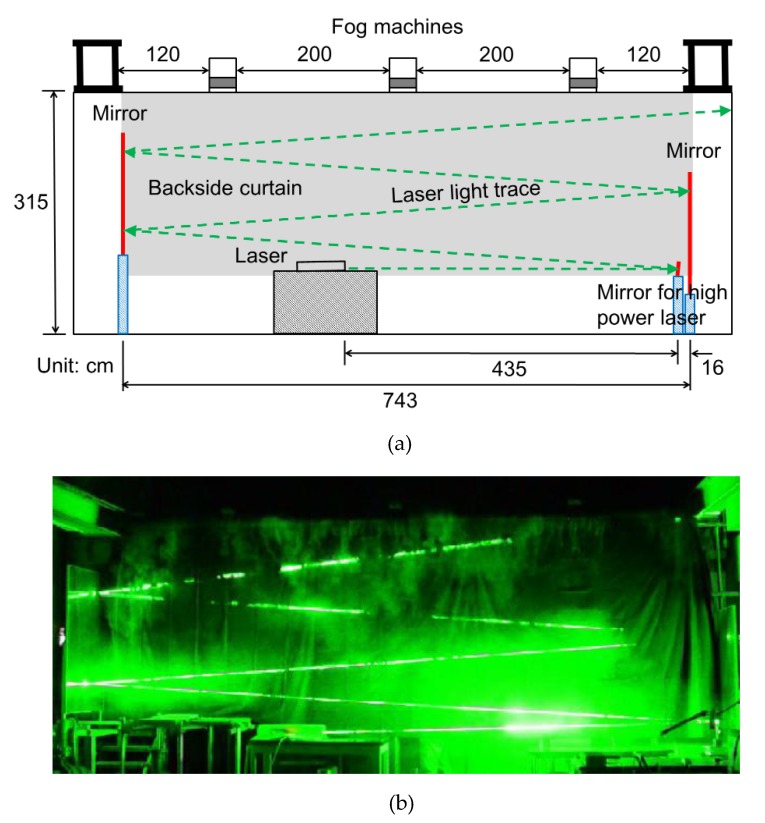
Front-side experimental set-up and a frame with a laser beam taken by a consumer video camera (**a**) The set-up includes three fog machines on the stage, a pair of mirrors, a small mirror for high-power laser beam reflection and adjustment of the direction of the laser beam, a laser and a backside curtain. The camera and timing controllers are placed on the other side. (**b**) The frame is extracted from images taken with a consumer video camera. Other frames are completely dark. Fog released at the second emission is falling over the stagnated fog generated after the first release.

The experimental setup is shown in Figure 2a. A wooden stage, 3.15 m high and 7.22 m wide, was assembled. The stage had 2.2 m long drooping black curtains. A pair of mirrors of 1.62 m high and 0.6 m wide were erected with a horizontal distance of 7.43 m. Therefore, the width of the backside black screen was 7.43 × 2.20 m. Light travels 30 m in 100 nanoseconds for 10 frames at the frame interval of 10 ns. The laser beam was tilted by 4 degrees, and reflected by the mirrors four times, making the laser beam pass more than 30 m. The test camera was placed at a distance of 14 m from the stage. Three fog machines were placed on the stage and manually operated. Therefore, the timing between the generation of smoke and operations of the electronic devices was controlled by oral communication. Details of the timing control and the fog generation is described in Section 2.3.

### 2.3. Timing Control

Operation of cameras with silicon image sensors is simple and flexible, which is the reason why they have overwhelmed other imaging devices. However, trial imaging was repeated, since the timing control of the fog generation and the camera system operation was not easy. We finally employed a two-stage spreading method of the fog.

After 62 s from the first fog emission for 10 s, the fog stagnated up to the height of our heads by thermal stratification in the laboratory, while the space above the height was still clear. Then, a second emission was added for 3 s to cover the upper half of the backside black screen, with fog falling down across the space over the stagnating fog. Two seconds before the second emission, the fog machine handler orally ordered warming-up operation of the laser, start of the automatic operation of the timing generator, and turn-off of the ceiling lamps. Five seconds after the stop of the second emission, the timing generator released the image capture signal, which was delivered to the laser and the camera through the digital delay generator with proper delays. The total duration was 80 s.

### 2.4. Captured Image

**Figure 3:**
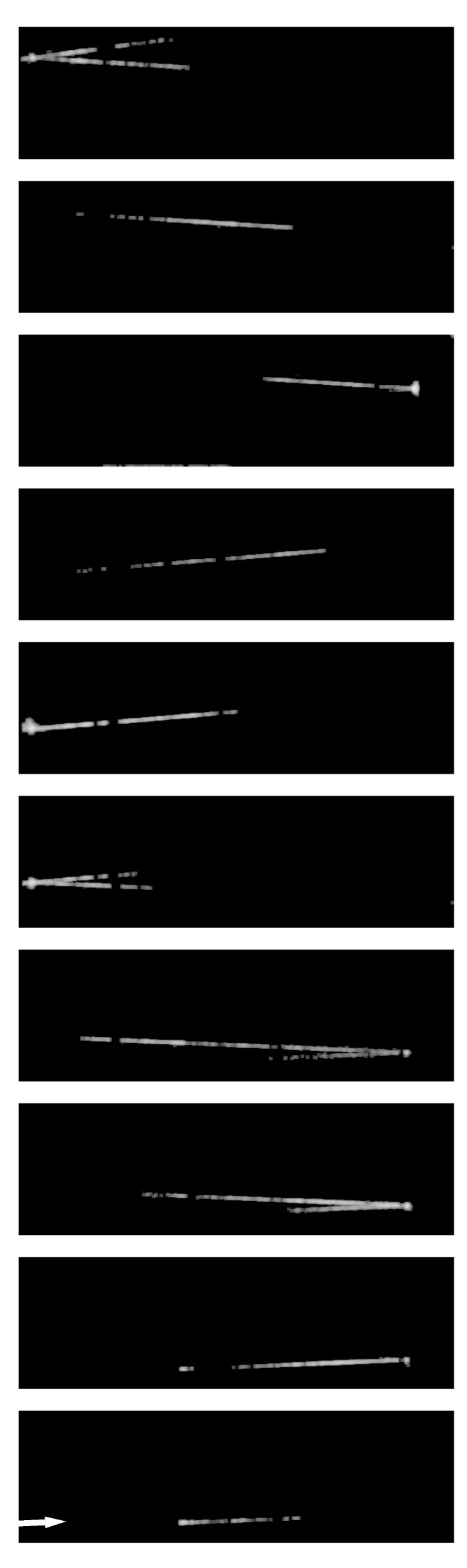
Images of the travelling laser pulse captured at the frame interval of 10 ns (from the bottom to the top). Much higher spatial resolution is achieved by the silicon image sensor than most of past light-in-flight imaging, except holographic images by Kubota and Awatsuji [2,3].

Figure 2b shows a laser beam image appearing in the fog distribution in one of the continuous frames captured by a consumer video camera. The other frames were all black. A laser beam with the half-value width of 5 ns was captured by the camera with the test sensor. Figure 3 shows the images of the travelling pulsed laser beam captured at 10-ns intervals. This is the first single-shot motion picture of the flying light captured by a silicon image sensor. A high spatial resolution as a single-shot flying light image was achieved with the image sensor of 300 kpixels. 

Each trajectory was about 3 m long. The shorter ones were due to locally faint fog. The longer ones resulted from effects in combination of the following factors:

(1) Blooming (overflow of signal charges from the pixels) at the local thick fog, especially near the mirrors, where the fog may have stagnated, or damage of the mirrors which may have caused additional strong emission of light;
(2) the overlaps of the driving voltages, and;
(3) tails before and after the main segment of the laser beam of the half-value width of 5 ns.

## 3. Suppression of Horizontal Motion of Signal Electrons for Ultimate-High-Speed

### 3.1. Surpression of Horizontal Motion of Signal Electrons

Innovative image sensor structures toward the ultimate high-speed imaging have been proposed and investigated by the theoretical analysis and simulations [12,13,15]. The most critical issue to achieve the ultimate high-speed is suppression of the horizontal motion of signal electrons. Hereafter, our works on the suppression of the horizontal motion are summarized with new proposals and analyses for further increase of the frame rate as follows:

(1) The theoretical temporal resolution limit
  By assuming the perfect suppression, the authors derived an expression of the theoretical temporal resolution limit of photo-conversion layers. The high accuracy of the expression was proved by comparison with the results of Monte Carlo simulations of the motion of signal electrons in silicon image sensors. The theoretical temporal resolution limit of silicon image sensors is 11.1 ps [15]. In this paper, the very high accuracy of the approximate expression is confirmed in comparison with the numerical calculation results of the strictly formulated expression that cannot be expressed with elementary functions.
(2) Practical methods for the suppression of the horizontal motion:
  A frame interval of 50 ps can be achieved by a silicon image sensor with a silicon pipe in the middle of each pixel to suppress the horizontal motion of generated electrons [13]. While the pipe can be fabricated by an existing technology, the sensor requires a light focusing device such as a micro-lens or light-guide array on the backside. In this paper, a convex silicon pyramid is proposed for charge collection, which eliminates the requirement and will provide an image sensor with the time resolution better than 100 ps with 100% fill factor.

### 3.2. Comparison of the Approximaate Expression of Theoretical Highest Frame Rate with Numerical Calculation of the Strictly Formulated Expression

**Figure 4:**
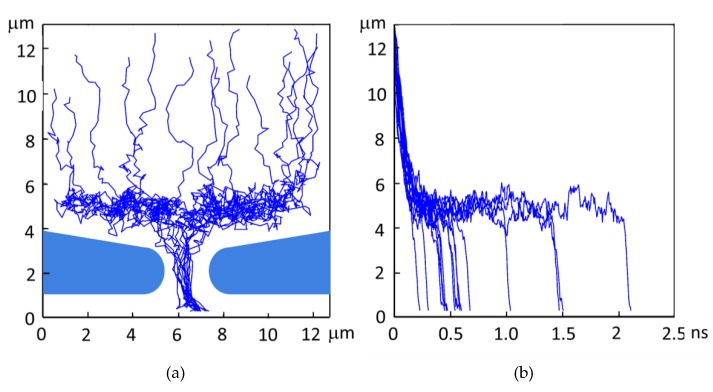
Charge collection by the p-well. (**a**) Trajectories of signal electrons calculated by a Monte Carlo simulation method. (**b**) Travelling time (x-axis) vs. travelling distance from the backside (y-axis). The p-well has two functions: potential separation of the upper signal generation layer and the lower circuit layer, and charge collection to the center of each pixel. For an ultra-high-speed operation, the size of the collection gates should be minimized to reduce the capacitance load. Therefore, the signal charges should be collected at the center. As shown in (**b**), the major cause of spread of the arrival time, i.e., the temporal resolution, is the horizontal motion of signal electrons moving toward the pixel center over the p-well. The temporal resolution for this case analyzed by simulations are shown in the second column of Table 2.

Temporal resolution is dependent on the distribution of the arrival time of signal electrons. Figure 4 shows example trajectories of generated electrons and the relation between the travel time and the travel distance from the backside for a BSI MCG image sensor with the potential separation by the p-well. As shown in the figure, the major cause spreading the arrival time is the horizontal motion of signal electrons travelling over the p-well to the center of the pixel. 

A silicon pipe with an infinitesimal diameter perfectly suppresses the horizontal motion. The remaining vertical motion has two governing factors: mixing of electrons due to the exponential distribution of the penetration depth of light, and pure diffusion due to the vertical random motion of generated signal charges. Based on the assumption, the temporal resolution of photoelectron conversion layers, including silicon layers, was theoretically analyzed. Fortunately, a simple approximate expression of the theoretical temporal resolution limit was derived. The accuracy was confirmed in comparison with the temporal resolution calculated by Monte Carlo simulations [15,16]. The expression of the travel time distribution can be derived with no approximation, while it cannot be expressed by elementary functions. In this paper, our approximate expression is compared with the numerical calculations of the strictly derived expression. Almost perfect agreement is confirmed by the comparison for a range used in practical applications.

Figure 1 shows the superposition of distribution functions of two signal packets generated by double instantaneous illuminations to the backside of the sensor and dispersed during the travel, where Δt is the interval of the double illuminations, and σ is the standard deviation of the arrival time of signal electrons at the collecting gate on the front side. If a Gaussian distribution is assumed as the arrival time distribution, for Δt > 2σ, a dip appears at the center of the superposed distribution. Therefore, the no-dip condition, Δt = 2σ, was employed for the separability criterion for the temporal resolution [15]. This is a similar concept to the Rayleigh’s criterion for the spatial resolution applied to the superposed Airy’s diffraction patterns with a 16% dip at the center [14].

The expression of the arrival time distribution can be derived through a strict theoretical analysis. However, the resultant expression cannot be expressed with elementary functions, requiring numerical calculations to observe the characteristics. A common method to obtain an approximate expression from a rigorous analytical expression is to expand the original expression to a series under a specific condition and employ the lower order terms. However, we employed a different approach. The arrival time distribution asymptotically approaches the Gaussian for a large *W* or a large *D*, where *W* is the thickness of the photoelectron conversion layer, and *D* is the vertical diffusion coefficient. By assuming the Gaussian distribution at the arrival section, we derived an explicit approximate solution of the temporal resolution limit, which is two times of the standard deviation of the arrival time, as follows [15]:

(1) $$t_{A}=\;2\sigma_{sA},$$

where

$$\sigma_{sA}^{2}=\sigma_{m}^{2}+\sigma_{d}^{2},\;\sigma_{m}^{2}=\left\{-{W^{\prime }}^{2}\left(1-p\right)/p^{2}+1\right\}t{}^{\prime }{}^{2},\;\sigma_{d}^{2}=\left(D^{\prime }t^{\prime }\right)\left(W^{\prime }-p\right)/p,$$

$$p=\int_{0}^{W}k\left(s\right)ds=1-\exp\left(-W/\delta \right)\;=1-\exp\left(-W^{\prime }\right),$$

(2) k(s)=(1/δ)exp(−s/δ) ,

where $\;\sigma_{sA}^{2},\sigma_{m}^{2}\;\mathrm{and}\;\sigma_{d}^{2}$ denote respectively the approximate expression of the variance of the arrival time and those caused by the mixing effect due to the penetration distribution k(s) of photons and the pure diffusion effect due to the random motion of generated electrons; $W^{\prime }=W/\delta ,\;t^{\prime }=\delta /v,\;\mathrm{and}\;D^{\prime }=2D/v^{2};$ *δ* represents the average penetration depth and v representsthe drift velocity.

When the values of the four parameters, *W*, *δ*, v and *D* are assigned, the temporal resolution limit Δt_A_ is calculated. The values of *δ*, v and *D* are dependent on the wavelength of the incident photon, the material and the environmental conditions including the electric field, temperature and pressure.

Equation (1) provides not only the expression of the temporal resolution limit, but also examining the conditions of these parameters to decrease Δt_A_ leads to the sensor structure which minimizes the temporal resolution limit. For example, the value of the drift velocity v saturates and the vertical diffusion coefficient *D* takes the minimum value at the critical field 25 kV/cm, which minimizes Δt_A_. The crystal orientation <111> of the Silicon layer provides slightly smaller *D* than the <100> layer, while the availability of the <111> wafer is low in practice.

Factors governing the temporal resolution limit are apparently observed from the simple expression of Equation (1). The parameters, $W{}^{\prime }$, $t^{\prime }$ and $\;D{}^{\prime }t{}^{\prime }$ are standardized with the average penetration depth δ, respectively representing the thickness, the drift duration, and the diffusion per the average penetration depth. 

The unit drift duration $t^{\prime }$ is a dominant factor; for $t{}^{\prime }\gg 1\;\mathrm{and}\;t{}^{\prime }\ll 1$, the mixing and the diffusion respectively governs the temporal resolution. By taking the limits of the parameters, Equation (1) is reduced to simple expressions, which describe the basic characteristics of the temporal resolution limit.

To examine the accuracy of Equation (1), the expression of the temporal resolution limit is derived with the strictly theoretical formulation. The one dimensional spatial distribution at a time *t* and a depth *z* of an electron generated at *t* = *z* = 0 is expressed by a Gaussian distribution [21]:

(3) $$g\left(z,\;t\right)=\left(1/\sqrt{4\pi Dt}\right)\exp\left\{-{\left(z-vt\right)}^{2\;}/\left(4Dt\right)\right\}\;$$

However, the temporal distribution passing through *z* = *W* skews from the Gaussian distribution with a slightly acute front and a longer tail, since electrons arriving at *W* earlier and later are respectively affected less and more by diffusion. The temporal distribution, i.e., the flux distribution at *W*, is derived by inserting the spatial distribution, Equation (3), to the drift diffusion equation as follows:

(4) h(z, t)=vg(z, t)−Ddg(z, t)/dz=1/2(1+z/t)g(z,t)

Electrons generated at the depth *s* travels (W−s). The flux distribution of the electrons at *W* is expressed by inserting the travel distance (W−s) to *z* in Equation (4). The probability distribution of the flux weighted by the distribution of the penetration depth is the product of Equation (2) and Equation (4). The total flux distribution is the integration of the product between (0, W) with respect to *s*.

(5) $$f\left(t\right)=\int_{0}^{W}k\left(s\right)h\left(W-s,\;t\right)ds$$

The 0th, 1st and 2nd moments of the arrival time with respect to *t* are as follows:

$$p=\int_{0}^{\infty }f\left(s\right)ds,\;\;E\left(t\right)=\int_{0}^{\infty }t\;\left\{f\left(s\right)/p\right\}ds,\;E\left(t^{2}\right)=\int_{0}^{\infty }t^{2}\left\{f\left(s\right)/p\right\}ds$$

The temporal resolution limit is:

(6) $$\Delta t_{E}\;=2\sigma_{sE},$$

where

$$\sigma_{sE}^{2}=E\left(t^{2}\right)-{\left\{E\left(t\right)\right\}}^{2}.$$

Equation (6) is formulated in the strict manner. However, it cannot be expressed with elementary functions. The value is numerically calculated.

The approximate expression Δt_A_ is compared with the numerical value of the exact solution Δt_E_. For the comparison, an intrinsic silicon layer of the crystal orientation <111> is assumed, which provides a lower drift velocity v and a larger diffusion coefficient *D* than those of a <100> silicon layer, resulting in a shorter temporal resolution limit. In this case, the values of v and *D* are respectively 9.19 × 10^6^ cm/s and 10.8 cm^2^/s at 300 K under the critical electric field of 25 kV/cm [15,22,23]. The wavelength of the incident light and the energy of the incident X-ray are assumed 550 nm (green light, 2.25 eV) and 10 keV, for which the average penetration depths are respectively 1.733 μm [24] and 126.6 μm [25]. As the wavelength of visible light is between 400 nm and 700 nm, light with the wavelength of 550 nm was selected as a representative visible light. 

**Figure 5:**
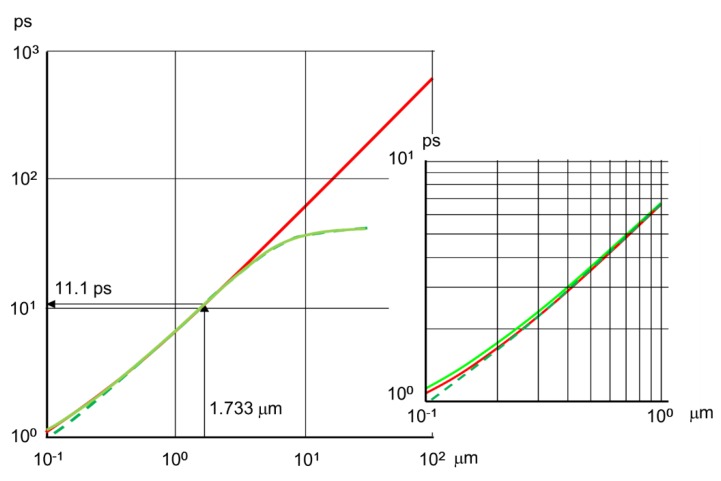
Comparison of the approximate expression of the temporal resolution limit Equation (1) with the numerical calculation of the strict expression (the exact solution) Equation (6). Green solid lines: λ = 550 nm, strict expression Equation (6); green dashed lines: λ = 550 nm, approximate expression Equation (1); red solid lines: X-ray, 10 keV, strict expression Equation (6); red dashed lines, X-ray, 10 keV, approximate expression Equation (1) (invisible due to overlap with the strict expression). The temporal resolution limit is 11.1 ps for a silicon layer <111> and *W* = *δ*, receiving incident light of 550 nm (penetration depth: 1.733 μm) under the critical field of 25 kV/cm and 300 degrees Kelvin.

The results are shown in Figure 5. The approximate expression perfectly agree with the numerically calculated exact solution for W>0.4 μm both for green light of 550 nm and an X-ray of 10 keV. The range covers the values used in practice. Furthermore, for 0.4 μm<W<3 μm for the green light and 0.4 μm<W<300 μm for the X-ray, the temporal resolution limit is approximated by the following relation within the 1.5% error:

(7) Δt=2σ=0.589 W/v =6.41 W

where v=0.0919 μm/ps for the critical field, and the values of Δt and W are in ps and μm. The values of the constants in Equation (7) are slightly different from Equation (11) in the prior paper [15]. The reason is that the latter one was derived by omitting the second term in the square root in Equation (10).

When $W^{\prime }=1$, i.e., the thickness *W* is equal to the average penetration depth *δ*, the temporal resolution limit for the representative visible light of 550 nm estimated from Equation (1) is compared with the exact solution calculated from the strict formulation Equation (6). The values are respectively 11.108 ps and 11.119 ps. The difference is only 0.1%. The temporal resolution limit, 11.1 ps, is reconfirmed by the exact solution. The theoretical highest frame rate is the inverse, 90.9 Gfps.

If a BSI silicon image sensor is designed by strictly following the conditions introduced in the theoretical analysis, the sensor will achieve the theoretical temporal resolution limit. However, some of the conditions conflict with other performance parameters of silicon image sensors, such as sensitivity and crosstalk. The temporal resolution 2σ represents the limit for the non-dip condition. In practice, the frame interval of 3σ to 4σ sufficiently suppresses the temporal cross talk. When $W^{\prime }=W/\delta =3$, instead of 1, the absorption rate (sensitivity) p= 95.0%, and the crosstalk due to photons remaining after the absorption is reduced to a practically negligible level.

The parameter values in Equation (1) are selected, depending on applications. For the high-speed X-ray image sensor developed by Claus et al. [26], $W^{\prime }$ is around 1, since the circuit layer on the front side is much thinner than the penetration depth, causing less crosstalk due to electrons generated in the circuit layer, while the signal generation layer should be thick enough to keep a reasonable absorption ratio.

### 3.3. Suppression of Horizontal Motion of Electrons with Convex Pyramid Charge Collector

A narrow square silicon pipe is assumed, where both the incident light and generated electrons are guided to the front side, and disperse at the bottom end of the pipe. This pipe architecture can be implemented by vertical etching of a silicon surface with the crystal orientation <100>, which is a well-known existing technology. The pipe was named a light-charge guide pipe (LCGP). 

The efficiency for suppression of the horizontal motion was evaluated through simulations by changing the diameter and the length to adjust the tradeoffs between the frame rate, sensitivity and crosstalk. The critical field is 25 kV/cm. The result of the practical optimization is shown in the third column of Table 2, where the temporal resolution of 49 ps is achieved. 

**Figure 6:**
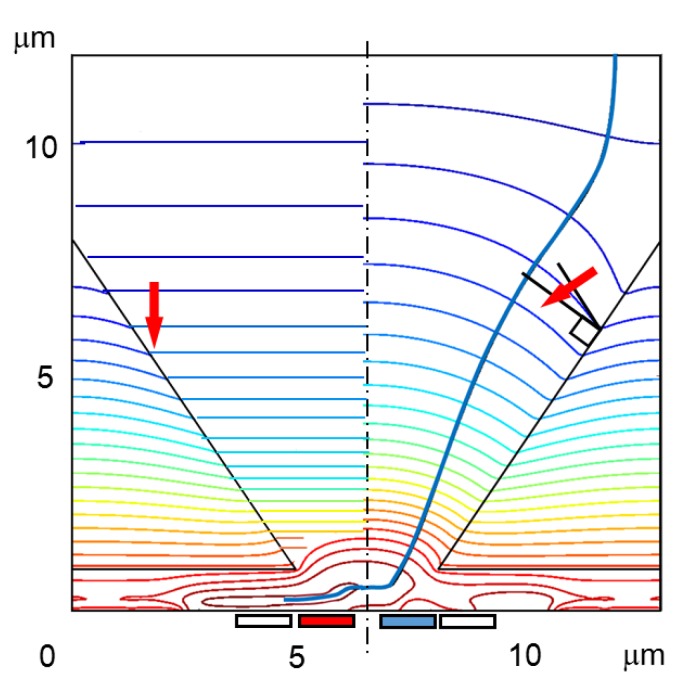
A convex pyramid charge collector. The potential is designed for the equi-potential contours to cross the surface of the pyramid with more than 90 degrees (the right half). The blue line: an example electron trajectory. The temporal resolution limit is 87.5 ps as shown in the fourth column of Table 2. The fill factor is 100%. The structure may be the ultimate one for ultra-high-speed imaging. It is especially suitable for ultra-high-speed X-ray imaging. Technology to make a high-quality convex silicon pyramid should be developed.

Even though the LCPG can be made with an existing technology, it requires an effective light focusing component attached on the backside, in spite that the major advantage of the backside illumination is the 100% fill factor. Hence, we will propose a convex silicon pyramid as shown in Figure 6. A <111> silicon surface appears by etching the <100> surface with an angle of 54.7 degrees under an appropriated condition. With the technique, concave and convex silicon pyramids can be formed [27,28,29,30,31]. The field in the direction along the pyramid surface is 81.6% (sin 54.7 degrees) of the vertical one. Therefore, it is expected that a temporal resolution may be close to the resolution achieved by the LCPG. 

An array of concave silicon pyramids (pyramid-shaped holes) have been applied to solar cells to reduce the reflection factor at silicon surfaces [28,29]. Yokogawa et al. applied the concave silicon pyramid array to their infrared image sensor to decrease dark current by enhancing diffraction of incident light with the pyramid array and making the silicon layer thinner [31]. Before the application, they improved the quality of the concave pyramid array to sufficiently suppress dark current from the pyramid array. While, at this moment, a high quality convex pyramid array is not available, if a good application is presented, it will not take a long time to develop a technology to improve the quality.

Apart from the process technology, a crucial problem associated with the convex pyramid array is how to guide signal electrons to the outlet at the bottom, avoiding collision of the electrons to the pyramid surface. The crossing angle between the equi-potential contours and the pyramid surface must be more than 90 degrees. Then, electrons move inward in the pyramid. A simulation study is performed to confirm the technical feasibility of the structure. 

The thickness of the total silicon layer of the simulation model is 13.1 μm, consisting of the backside hole accumulation layer of 0.1 μm, the generation layer of 12.0 μm (three times the penetration depth 4 μm of 650-nm red light), and the circuit layer of 1.0 μm. The pixel is a 12.73 μm square. The critical field of 25 kV/cm is applied to the generation layer.

A thin Boron layer is applied over the pyramid surface, and a small circular Phosphorous implant is introduced at the center of the outlet of the pyramid. Then, concentrations of the dopants was adjusted by simulations to increase the electron collection ratio (the number of electrons collected by the collecting gate/the number of generated electrons). The resultant potential field is shown in the right half of Figure 6, which collects more than 98% of the generated electrons as shown in the fourth column of Table 2. Furthermore, the sensor can achieve the ultimate high signal-to-noise ratio (S/N). The fill factor is 100%, and the photo-conversion rate can be more than 90% for *W* = 3*δ*.

**Figure 7:**
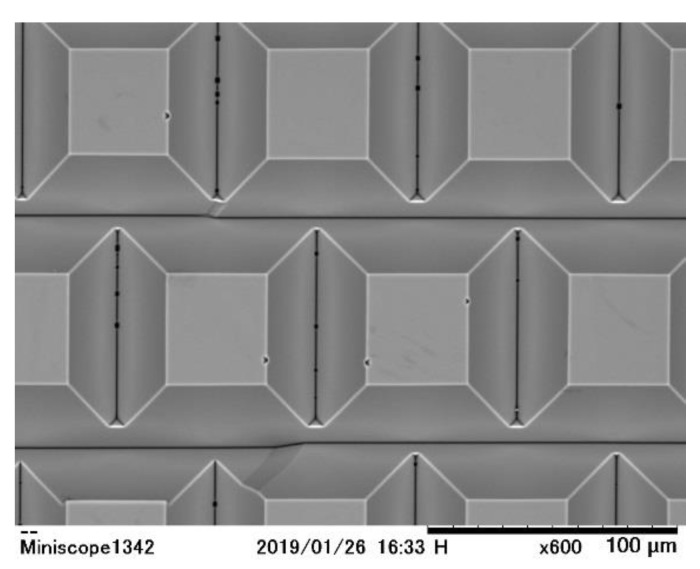
A convex pyramid array fabricated as a preliminary test by T. Ando.

Figure 7 shows a convex silicon pyramid array fabricated by Ando. This is a preliminary one with a large size due to the limited performance of the MEMS facility of Ritsumeikan university. Still, it proves technical feasibility of the technology not only based on simulations shown in Figure 6 and Table 2, but also on a physical experiment. Further research on the fabrication technology is necessary, especially, for stacking a monocrystalline silicon layer to the top of the pyramids. One possible method may be the Si–Si direct bonding with high-temperature annealing.

The pyramid funnel has a huge application potential for BSI global shatter image sensors, 100% fill factor ultra-fast image sensors with in-pixel memories, detectors for imaging TOF MS with direct ion or electron bombardment on the backside, a device to connect a bundle of optical fibres with a Silicon or Germanium detector array for ultra-high-speed communication, and so on.

The size is too large at this moment due to limitation of our MEMS facility; the top of each pyramid should be shrunk more; a silicon layer is stacked on the top and the circuit is fabricated in the layer.

## 4. Further Evolution of BSI Multi-Collection-Gate Image Sensors

### 4.1. Pipeline Operation for More Frame Count and Signal Accumulation

The multi-collection-gate structure achieves ultra-high-speed multi-framing. However, the frame count is equal to the number of collection gates, which is less than eight to avoid crosstalk due to migration of electrons to the neighboring collection gates. A memory circuit with multiple memory elements attached to each collection gate will solve the problem. A test sensor with four collection gates each connected to a four-phase CCD memory with 305 elements was proposed [18], fabricated and evaluated [19]. The frame count of the sensor is 1220 frames (305 × 4). The combination of the four collection gates each connected to a four-phase transfer CCD perfectly allows the pipeline operation. CMOS circuits for in-pixel signal accumulation were also proposed [32].

### 4.2. Macro-pixel Image Sensor 

A macro-pixel image sensor consists of an array of macro-pixels, each with an array of element pixels, such as 2 × 2 or 3 × 3 pixels [15]. The macro-pixel image sensors provide another way of ultra-high-speed imaging by capturing consecutive images with the element pixels in turn. Mochizuki et al. achieved the frame intervals of 5 ns by combining the structure with an advanced post data process [33]. Claus et al. achieved the frame interval of 2 ns for two consecutive frames, and increased the frame count to eight frames with the macro-pixel operation [26]. However, the sensitivity of the macro-pixel image sensors decreases to 1/N, while the frame count increases to N times, where N is the number of element pixels. A combination of the BSI MCG and the macro-pixel structures may be the most promising structure for ultra-high-speed imaging. Our 10-ns image sensor also exploits the advantage for the interlace operation, where N = 2.

### 4.3. Driver 

A dedicated driver circuit named “ROXNOR circuit” to drive the collection gates of the BSI MCG image sensor was proposed for 3D stacking of the sensor and the driver chips. Test chip has been fabricated and evaluated. The driving pulse of 1 ns wide and 3.3 V high has been achieved [34]. A circuit simulation shows that the pulse width can be decreased to 200 ps by decreasing the driving voltage amplitude of the sensor to 2 V.

The BSI MCG image sensor and the ROXNOR driver were fabricated with a 130 nm process. If a 65 nm process is applied to their design and fabrication, the capacitance load is significantly reduced, which will realize the silicon image sensor operating at 100 ps or less.

## 5. Conclusions

Evolution of ultra-high-speed image sensors is reviewed with new proposals and analyses.

(1) The-state-of-the-art ultra-high-speed image sensor
  A silicon image sensor achieved the temporal resolution of 10 ns. Light in flight is captured with the image sensor.
(2) The theoretical temporal resolution limit
  The most critical issue for increasing the frame rate is suppression of the horizontal motion of signal electrons. Assuming the perfect suppression, an approximate expression of the theoretical temporal resolution limit was derived. The very high accuracy of the expression is confirmed in comparison with numerical calculation results of the expression rigorously formulated. The theoretical limit for silicon image sensors is 11.1 ps.
(3) The practical temporal resolution limit
  The convex pyramid charge collector is the most promising method to effectively suppress the horizontal motion, which achieves the temporal resolution of 100 ps, keeping the 100% fill factor.

## Figures and Tables

**Table 1:** Specifications of the test BSI MCG image sensor [13] *.

Structure	BSI MCG Image Sensor
Shortest frame interval (Equivalent frame rate)	10 ns (100 Mfps)
Frame and pixel counts	5 frames for 576 × 512 × 2 pixels ** 10 frames for 575 × 512 pixels
Fill factor	100%
Charge handling capacity	7000 e^−^
Pixel size	12.73 × 12.73 μm (Diagonal 18 μm)
Photoreceptive area	10.368 × 9.216 mm
Process	130 nm CMOS process modified for CCD

* Backside-illuminated Multi-Collection-Gate image sensor ** Staggered pixel configuration: pixels on 512 odd-numbered columns are vertically shifted at a half pixel pitch to pixels on 512 odd-numbered columns.

**Table 2:** Structures for charge collection and potential separation for BSI image sensors. Pixel size, 12.73 μm; thickness, 13.1 μm, voltage amplitude to drive collection gates, 2 V; width of the light-electron guide pipe (square), 4 μm; outlet of the pyramid (square), 3 μm.

Structure	p-Well	Light/Electron Guide Pipe	Convex Pyramid Charge Collector
Cross sections	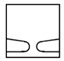	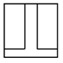	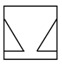
Temporal resolution 2σ	990.0 ps	49.0 ps	87.5 ps
Vertical Field	5 kV/cm *	25 kV/cm *	25 kV/cm *
Collection Ratio ***	100%	100%	98%
Dark current	less	middle	large
X-ray	Applicable	Low efficiency	Ideal
Technical feasibility	Already applied	Existing technology	Process improvement
Requirement	Linear built-in potential ****	Micro lens/light guide necessary	High-quality convex pyramid unavailable

* A field higher than the value causes a punch-through of the p-well. If a higher concentration of the p-well for a stronger potential separation is employed, it degrades performance of MOSFETs on the front side. ** The critical field minimizing the temporal resolution. *** Number of electrons collected by the collecting gate/number of generated electrons **** Minimizing horizontal travel time.

## Supplementary Materials

Supplementary File 1
